# A Stochastic Process Model for Free Agency under Indeterminism

**DOI:** 10.1111/1746-8361.12222

**Published:** 2018-08-24

**Authors:** Thomas Müller, Hans J. Briegel

**Affiliations:** ^1^ Fachbereich Philosophie Universität Konstanz Fach 17 78457 Konstanz Germany; ^2^ Institut für Theoretische Physik Universität Innsbruck Technikerstraße 21a, 6020 Innsbruck, Austria; Fachbereich Philosophie, Universität Konstanz, Fach 17 78457 Konstanz Germany

## Abstract

The aim of this paper is to establish that free agency, which is a capacity of many animals including human beings, is compatible with indeterminism: an indeterministic world allows for the existence of free agency. The question of the compatibility of free agency and indeterminism is less discussed than its mirror image, the question of the compatibility of free agency and determinism. It is, however, of great importance for our self‐conception as free agents in our (arguably) indeterministic world. We begin by explicating the notions of indeterminism and free agency and by clarifying the interrelation of free agency and the human‐specific notion of free will. We then situate our claim of the compatibility of free agency and indeterminism precisely in the landscape of the current debate on freedom and determinism, exposing an unhappy asymmetry in that debate. Then we proceed to make our case by describing the mathematically precise, physically motivated model of projective simulation, which employs indeterminism as a central resource for agency modeling. We argue that an indeterministic process of deliberation modeled by the dynamics of projective simulation can exemplify free agency under indeterminism, thereby establishing our compatibility claim: Free agency can develop and thrive in an indeterministic world.

## 1 Introduction

Can there be free agency, a capacity exemplified by humans as well as by many non‐human animals, if the world is indeterministic? This question is arguably more important than its mirror image, the question whether free agency is compatible with determinism. But unlike that latter question, the compatibility of free agency and indeterminism is not often addressed explicitly. In this paper we argue that free agency and indeterminism are compatible. We make our case by providing an explicit model in which an indeterministic, stochastic process – a random walk – is constitutive of the behavior and development of a free agent over the course of time.

Providing an explicit model is important for three reasons. First, in giving such a model, we show that, and how possibly, there can be free agency in an indeterministic world. Since there are good reasons to assume that our world is indeterministic, this should be good news for all defenders of human freedom. Second, in our model, indeterminism turns out to be a valuable resource that free agency can be based on, rather than a disturbing influence as often assumed. This provides a fresh perspective on the role of indeterminism in action. Third, in stressing the importance of a positive ‘how possibly’ argument for the compatibility of free agency and indeterminism, we hope to help to clarify the dialectics of the debate about freedom and determinism.

The structure of our paper is as follows. We start, in section 2, by clarifying the basic concepts of indeterminism and free agency. We then describe the dialectics of the free agency/determinism debate and thereby situate our paper in the context of a broader discussion. In section 3, we describe a stochastic process model for free agency under indeterminism, based on the AI learning scheme of projective simulation. We explain how the short‐ and long‐term dynamics of the model provide the basis for flexible, adaptive, and sensible agency, discussing a concrete example in section 4. We conclude in section 5.

## 2 The dialectics of free agency and (in)determinism

### 2.1 Basic concepts

In this paper we address the interrelation of two important concepts, one action‐theoretical – free agency – and one metaphysical – determinism. Before we enter a discussion of their dialectical interrelation, we clarify our use of these two concepts.

We need to stress up front that there is a considerable difference in the basis on which our characterizations of these two crucial notions rest. *Determinism* is a philosopher's term of art that has a fairly well‐defined meaning. While the term has somewhat varying uses in the current freedom debate, its meaning is historically quite stable, due to determinism's role in an unbroken tradition of arguments over centuries. Therefore, in section 2.1.1 we can simply clarify our specific use of the term with respect to a few nuances that have played a role in extant debates. *Free agency*, on the other hand, is a notion whose importance for various philosophical discussions has only become apparent with the advent of action theory as a philosophical sub‐discipline in the second half of the 20th century, and terminology is not established firmly. Most of the current freedom debate, including its terminology, focuses on free will rather than on free agency. Our focus is, however, on free agency, which is one of the preconditions of free will, and therefore more basic. A discussion of free will arguably leads beyond the realm of action theory proper, and free will may well be an exclusively human capacity. Free agency, in contrast, should apply to human beings, many animals, and possibly also (future) artifacts. 1There is a growing literature that promotes ‘action theory first’ when it comes to metaphysical aspects of freedom. For a particularly good example, see Steward (2012). She also provides compelling cases of non‐human animal agency including, but not limited to, other mammals.


Our aim is to delineate a rich and philosophically useful concept that avoids two obvious pitfalls: triviality on the one hand, and philosophically loaded presuppositions that threaten to limit free agency to human beings, on the other hand. Accordingly, our clarificatory discussion of free agency in section 2.1.2 is not just a matter of taking a stance on nuances of extant use, but rather a philosophical act of explication. Since that explication may be controversial, we proceed slowly, step by step. But first, determinism.

#### 2.1.1 Determinism and indeterminism

To begin with the metaphysical side, by determinism we mean the metaphysical claim about our world that as of now, there is only one real possibility for the future to turn out. Indeterminism, on the other hand, is defined simply as the negation of determinism: as of now, there is more than one really possible way for the future to turn out. These definitions refer to the notion of real possibilities for the future, without specifying that notion any further. 2For a discussion of the notion of real possibility vis‐à‐vis other notions of possibility, see Müller (2012) and Rumberg (2016). In the literature, the future possibilities in question are often further specified to be possibilities that are allowed for by the laws of nature given the present state of the world. Thus, it is common to define (‘Laplacean’) determinism as the claim that given the current (maximal intrinsic) state of the world and the laws of nature, only one future development is compatible with these laws. 3See, e.g., Butterfield (2005) and Earman (2006). See Müller (2015) and Müller and Placek (2018) for discussion. In our view, this additional specification of the future possibilities in question amounts to an unnecessary and possibly harmful addition to the basic definition of determinism, which can and should be avoided in the debate about free agency. The notion of a current state of the world is philosophically highly contentious, 4If a state is not specified via intrinsic properties, a verdict of determinism can be spurious. For example, if you describe today as ‘a day before a week of rain’, which is an extrinsic characterization, then no alternative possiblity is left open for next week's weather. We need states to be intrinsic. In the current debate about intrinsic properties, however, no real consensus has been reached; see, e.g., Langton and Lewis (1998) and Eddon (2011). Considerations of the relativity of simultaneity may raise further worries in connection with the requirement of having to specify a global Now to make sense of a ‘current state of the world’. and similar problems surround the notion of a law of nature. 5Some respectable authors, such as Van Fraassen (1989) and Giere (1999), even defend the claim that in the relevant sense, there are no laws of nature. Note that given the definition of determinism in terms of laws and states, the absence of laws implies that the world is indeterministic, because *any* future development is compatible with the empty set of laws. In order to discuss the interrelation of freedom and determinism, no position needs to be taken with respect to the metaphysical status of laws of nature and intrinsic states. What is important is that according to universal determinism, at any time there is just one real possibility for the future. It is this idea of predetermination, of the uniqueness of the possible development of the world, which arguably spells trouble for the notion of freedom, independently of any account of laws and intrinsic states.

#### 2.1.2 Free agency

Turning to action theory, we will use the expression ‘free agency’ and related terms in the following way: Free agency is a capacity of certain beings, free agents. These agents act freely at least some of the time. We contrast a free action with non‐agentive, mere behavior. A case of behavior (typically, a bodily movement) thus is classified as mere behavior, or as free action, 6We avoid taking a stance on the thorny issue of whether some instances of mere behavior constitute actions, albeit unfree ones. There are good reasons to hold that agency, properly understood, has to be free agency, implying real alternatives. On the other hand, we do call some of our actions unfree, e.g., when we act under coercion. In fact, under the heading of ‘freedom of action’ one can find a vast literature on free vs. unfree actions (for an overview, see, e.g., O’Connor 2016). For our present purposes, we do not need to discuss cases of purported unfree actions. We stick to clear examples of free actions vs. mere behavior that do not involve coercion, political oppression, compulsive behavior or such like. and free agents are those beings that can act freely. 7We stress the importance of both the persisting entity, the agent, and the event in which the agent is involved, the action. It is true to say both that the agent causes certain happenings, and that these happenings are events. In this way, we avoid the dichotomy of ‘agent causal’ vs. ‘event causal’ approaches as unhelpful, following Kane (2014) in his ‘AC/EC’ approach. For example, human beings are free agents, and they typically act freely. Most of what we do – taking a sip of coffee, walking to the store, typing a sentence – exemplifies our free agency. We even exhibit our free agency when we intentionally refrain from moving, as in a game of hide‐and‐seek, or when we act sub‐intentionally, as in wiggling our toes during a boring meeting. Certainly there are boundary cases. But sometimes we also clearly exhibit mere, non‐agentive behavior – when a doctor triggers our patellar reflex by hitting us under the knee, for example, or when we blink when an object comes close to our eyes. We are free agents, but we do not exhibit our free agency all the time.

##### 2.1.2.1 Free agency vs. free will

Traditionally, agency and freedom are most often discussed under the heading of ‘free will’. There is a long discussion about the notion of free will itself as well as about its metaphysical preconditions. We will use some of the terminology that has been developed in that discussion, but in this paper we do not discuss free will. In philosophy, free will is mostly tied to specifically human traits, such as being the proper subject of moral praise and blame, or a capacity for conscious deliberation or for the linguistic expression of self‐reflective thought. 8See, again, O’Connor (2016) for a good overview of relevant philosophical issues. In the scientific literature, ‘free will’ is sometimes used less specifically than in philosophy – sometimes in the sense of our notion of free agency, but also for something even less demanding than that. For example, a ‘free will’ condition in quantum correlation experiments such as Hensen et al. (2015) has got nothing to do with morality, and in fact just demands the breaking of correlations with the past. In the biological literature such as Brembs (2011), ‘free will’ is used in a way that comes very close to what we here call ‘free agency’. Since this is a paper in philosophy, we stick to the usage of ‘free will’ established in that subject, which is also the historical origin of the notion. To be sure, these are central aspects of our human lives, and we do not want to diminish their importance in any way. By stressing the usefulness of a less demanding notion of free agency, we just want to oppose the idea that there is no sensible notion of freedom that avoids the specifically human commitments of the notion of free will. Our explication of the notion of free agency is meant to supply such a broader, more basic notion, which can also apply to other animals, and possibly also to (future) artifacts. 9A note on philosophical terminology to avoid a misunderstanding: There is a large discussion about so‐called ‘freedom of action’, meaning the absence of external obstacles (see note 2). What we are calling ‘free agency’ is not the same phenomenon as what is discussed as ‘freedom of action’. The latter notion is employed to illustrate or even replace the notion of ‘free will’ when it comes to assessing *human* behavior. We are aiming at a much broader notion. Also, ‘freedom of action’ is traditionally associated with making room for freedom under the assumption of determinism (so‐called compatibilism, see section 2.3.1), while we are looking for an indeterminism‐friendly notion of freedom, as explained in section 2.2.2. A clash between a notion of freedom and the doctrine of determinism (or of indeterminism) arises already for the thinner, less exclusive notion of free agency, not just for free will. 10On this point, see again Steward (2012). This important book has done a lot to show that the metaphysics of free agency is the proper place to discuss the issue of freedom vs. determinism. And however free will is spelled out in the end, free agency certainly forms a precondition for it. Thus we hold that a discussion of the interrelation of determinism and free agency has systematic priority over a discussion of the interrelation of determinism and free will, which occupies so much of the philosophical literature.

##### 2.1.2.2 Explicating free agency, via examples of attribution

Free agency is not limited to human beings, as we said: many animals are free agents. For example, cats and crows, when playing or looking for food, act freely. 11We again refer to Steward (2012, esp. ch. 4) for an abundance of examples. Methodologically, it is important to note that we are here after the *explication* of a notion of free agency for which there is no acknowledged off‐the‐shelf definition. This requires us to work out the boundaries of this notion starting from clear positive and negative cases, such as cats and crows vs. tables and chairs. This does not mean that we have to attribute free will to them, or to deny them freedom altogether when we find, quite sensibly, that they fall short of possessing free will. But neither would it be enough to attribute their behavior to them in a merely causal way. If a crow catches a worm, causal attribution is warranted, but what goes on is relevantly unlike a stone breaking a window. What we are trying to explicate is a notion of free agency that allows for the *attribution of an action to an agent* in a way that lies between mere causal attribution and the demands of attributing an action out of free will. The former type of attribution does not distinguish a free action from a case of mere behavior: behavior of both kinds is causally attributable. The latter type of attribution, on the other hand, traditionally implicates conscious self‐reflection and the possibility of moral assessment, limiting that type of attribution, for all that we know, to human beings.

In order to home in on the middle‐level notion of free agency that we are after, we start from the fact that we have a well‐entrenched practice of attributing agency in such a third, middle‐level way, both toward human beings and toward other animals. In fact, many of our everyday practices depend on that type of attribution. Consider, first, an example of human interaction: somebody stepping on your toes on a bus. In such a case we make a three‐fold distinction between (1) mere behavior, implying just causal attribution (e.g., a person stumbling because they were pushed and could not help it), (2) acting out of free will, implying moral attribution (e.g., a person acting with the morally blameworthy intention of hurting you, or with the morally praiseworthy intention of alerting you to a pick‐pocket), and (3) acting freely, but not out of free will, which implies non‐moral agency‐attribution (e.g., a person acting absent‐mindedly: behavior which was under their control and which they could have avoided, but which wasn’t intended either). Our reaction to what happens on the bus (e.g., whether we demand and/or accept an apology) depends strongly on these distinctions. 12In human interaction, moral categories may be brought to bear in all three cases when taking a longer‐term perspective, which is often adequate. This makes it difficult to give unassailable examples of ‘mere free agency’ attribution in humans. We expect others not just to refrain from hurting us intentionally, but also to take precautions against accidental damages, no matter whether these damages might come about through mere agency (absent‐mindedly, non‐intentionally) or even through mere causation (e.g., through reflexes). In almost all cases, our natural inter‐personal attitude is what Strawson (1962) has called the ‘reactive attitude’ of moral attribution. Thus, somebody may feel moral indignation toward a person who coughs during a concert, thereby diminishing their pleasure in the music. Here the coughing is a reflex and as such not subject to moral evaluation, but the person who coughs may have a cold and know that they are prone to coughing. (We do not take a stance on whether the moral assessment here is warranted. A lot usually depends on details.) Clearer examples of mere free agency attribution in humans occur, for example, in cases of physical training, as when you take a swimming lesson and the trainer provides feedback on the way you move.


Second, consider examples of feline agency. It may well be that for non‐human animals, acting out of free will is out of the question: we do not praise or blame a cat in a moral sense. But we do make a distinction between the cat's mere behavior and her free agency. In the former case, we view that cat, as it were, mechanically; in the latter case, we attribute actions to her as an agent that wants certain things and not others, and which can be influenced through teaching. We see this distinction clearly in cases in which the cat's behavior bothers us so that we want to stop it. 13This does not mean that the distinction has no place when the behavior pleases us, or when our attitude toward it is neutral. In fact, our attitude in the cases we describe might be different; this does not influence the type of attribution. Take a case of mere behavior, e.g., the cat shedding hair on your dark carpet. In order to stop that behavior, you may decide to brush the cat, or perhaps to get her on a diet leading to less shedding: the aim is to alter the cat physically in a direct way. In a case of the cat's free agency, our attitude is different. Thus, when the cat sits down on your keyboard while you are trying to type, you may recognize that this is a bad attention‐grabbing idea of hers, and try to teach her not to do it. Reacting in this way, you address the cat as an agent, as an individual who experiences the world and who can adapt her behavior over time when given feedback of certain kinds.

##### 2.1.2.3 Explicating free agency: criteria

Based on our remarks and examples so far, we can now attempt to explicate the notion of free agency in a more detailed way. We want to make its distinctive traits explicit with a view to assessing the model that we will present in section 3. As we said, defining free agency in a precise way is much harder than defining determinism. We will not argue for a fixed set of necessary‐and‐sufficient criteria here. We will, however, provide some necessary criteria and suggest a sufficient criterion.

We have already established that we attribute free agency in many cases, including cases in which we do not attribute free will, and that we dismiss the attribution of free agency in cases of mere behavior, which we attribute causally but not as agency. In building on these examples, we are assuming that the attribution of free agency tracks an important and real difference among what is going on in our world. The notion of a free agent (a system that can act freely) thus has clear instances, such as human beings, cats, and crows, and clear non‐instances, such as stones, tables, and thermostats, who exhibit mere behavior at best. 14It is possible to acknowledge the fact that we attribute free agency while denying that there is such a thing as free agency at all. Such a skeptical option (an error theory of agency attribution) is always available, but we can dismiss it as irrelevant for our approach, which aims at establishing the possibility of free agency by presenting an explicit model. Now, which difference in the world is it that the notion of free agency is tracking? What are the distinctive traits of free agency?

The main point flowing from our discussion so far is that an agent acting freely is special because it *does* something. It thereby influences the course of nature in a way that goes beyond mere causal influence: the action can be attributed to the agent *as an agent* since it was *up to the agent*, or *the agent was the source of the action*, or *the action was under the agent's control*. 15A sizable part of the free will discussion at present targets notions of control, such as regulative vs. guidance control (see, e.g., Fischer and Ravizza 1998). Since the examples used in discussions of control are often subject to moral assessment, and since there is no clear consensus on a taxonomy of different types of control or on which notion of control is needed for free agency or for free will, we do not frame our discussion in terms of control.


This thought has to be unpacked in order to yield a list of criteria. We start with a precondition of all kinds of behavior: (1) causal influence. This criterion is, however, not yet specific; causal difference‐making is not sufficient for free agency. Thermostats make a causal difference to what happens, but they cannot act freely. Further criteria have to be added. Guided by the above discussion, we propose the following minimal list of additional criteria: free agency has to be (2) non‐rigid, (3) flexible and adaptive, and (4) sensible. We proceed to argue for our criteria in turn, in the order of increasing strength.

1. Causal difference‐making is an uncontroversial precondition for agency. To act is to make a difference to what is happening. 16When discussing free will, it is important to count omissions and refrainings as morally attributable actions, at least in some cases. For example, we would morally blame a person for doing literally nothing when he could easily have saved a drowning child. We will not take a stance on the thorny issue of omissions in our discussion of free agency. It seems fairly obvious that we sometimes do attribute omissions as free actions, e.g., when a cat remains completely silent and immobile while preying on a mouse. But for our discussion in what follows, nothing hinges on this. Such difference‐making may be deterministic (as in a thermostat) or indeterministic (as in Feynman's (1965, 147) example of an indeterministic bomb). Neither deterministic nor indeterministic causation is yet sufficient for agency.
2. Free agents have the capacity to behave non‐rigidly. Rigid responses are typical of so‐called reflex agents. A thermostat is an example of this class: the response is always a function of the input (in the thermostat's case, the room temperature). A thing that has a unique, rigid way of reacting in any given situation is not a free agent. It is true that free agents, such as cats, do have a repertoire of rigid reflex behaviors, as we do ourselves. But free agents cannot be limited to reflexes in their behavior. 17There is a certain paradoxical aspect to rigidity vs. freedom: at least in the moral case, we often assume that an ideal free agent should have a firm character and always do the one right thing. But such an agent would behave rigidly. Would that take away her freedom? With respect to our list of criteria, it is important to stress that a free agent has to have the *capacity* to act non‐rigidly. This leaves room for rigid behavior in certain morally relevant situations. We take it to be an advantage of our approach that we can explain how adaptation through learning can lead to rigid behavior in selected circumstances, thus accounting for rigidity through freedom. See note 51 for some additional discussion. Note that acquired rigidity is not the same as a built‐in reflex. A reflex agent may have no capacity for acting non‐rigidly; a free agent who has acquired rigid behavioral traits has done so on the basis of learning from non‐rigid actions.
3. Requiring flexibility and adaptivity is a way of spelling out in which way a free agent should be non‐rigid. A thing that responds completely erratically, such as a lottery machine, is non‐rigid, but we would not call such a thing a free agent. For free agents, non‐rigidity plays a positive role on two different time‐scales. On a short time‐scale, free agents can act in a flexible way: in following through a course of action, such as moving from one place to another, a free agent has a repertoire of possible variants which are employed, e.g., to overcome obstacles. Finding a route blocked, a reflex agent may be stymied, while a free agent can vary its behavior and thereby find new ways to proceed. On a longer time‐scale, free agents adapt to their environment and the feedback they receive from it: they learn, and they can be trained (they may even be able to train themselves, through practicing and playing). Free agents develop over time, and the way they have developed influences the way they act at a given time. This means that for a free agent, the notions of identity over time and memory play a crucial role. Free agents have to be learning agents, whose history and experiences influence the state of their memory, and as a result, their behavior. For example, a hungry cat will do various things in order to find food (flexibility), and over time will learn where the mice live (adaptivity).
4. Our last criterion, sensibility, is a way of specifying in which way this flexibility and learning have to play out. The term ‘sensible’ is admittedly somewhat vague. The idea is that the flexibility and adaptivity of a free agent's actions have to be something *for* the agent, they have to *make sense* for it. In living beings, this idea can be explained in terms of the agent's *flourishing*, of satisfying its *needs*, and of its striving for what is *good* for it. It is a challenge, which we do not claim to have resolved fully, to spell out this idea of sensibility in a way that is general enough to apply uncontroversially both to natural and to artificial agents. It is certainly difficult to provide a foothold for the agent‐relative normative dimension involved here when it comes to artefacts. Agents can be sensible in different ways, so that the criterion of sensibility is inherently disjunctive. Rather than trying to spell out this full disjunction (which would provide a necessary criterion), here we spell out one type of sensibility as a *sufficient* criterion (without taking a stance on whether that criterion is also necessary): An agent fulfilling criteria (1) – (3) exhibits free agency if it acts on its own reasons or considerations, and develops the structure of its considerations (its memory) in a meaningful way over time. Note that again, two time‐scales are involved in this account: One is the short time‐scale of the flexible dynamics selecting from among the possible individual actions at a given time; the other is the long time‐scale of adaptation through learning from many individual actions. 18In the free will debate, the short time‐scale aspect of sensibility is stressed throughout, e.g., as a criterion of ‘reasons‐responsiveness’, which is often assumed to be compatible with determinism (see, e.g., Dennett 1984). The longer time‐scale of learning is much less discussed (but see Kane's view referred to in notes 47 and 51). In our view, the failure to acknowledge the long‐term perspective of learning is a significant shortcoming in discussions of free will, but even more so in discussions of its precondition, free agency.

##### 2.1.2.4 Notions explicitly avoided

In closing, we mention a few notions that we have explicitly tried to avoid in our explication of free agency. Our list of criteria does not mention intentions, nor the will, nor rationality, nor rightness or wrongness. We fully agree that these notions are important for a discussion of free will, and also for assessing human agency more generally. For our purposes, however, it is better to avoid them in order not to trigger misleading associations with the notion of free will.

It is clear that we need to avoid talking about rightness and wrongness – moral categories are not applicable when we limit ourselves to free agency. For intention, rationality, and the will, we do not take a stance on whether these notions can be fully accounted for on the basis of free agency alone. Rationality is sometimes tied to high‐level linguistic capacities, which would limit rationality to human beings; 19See, e.g., Davidson (1974). other uses of the term are less stringent. We do not wish to become tangled up in the associated terminological debate, and thus we avoid speaking of rationality. With respect to the will, the situation is similarly complex. There is an important tradition that holds the will to provide the direct target for moral assessment; 20See, e.g., Kant's *Groundwork of the Metaphysics of Morals* (Kant, 1785). and in this sense, the will has no place in our discussion. A less loaded notion of the will may, however, very well be appropriate in the discussion of free agency. A similar comment applies to the notion of intention: in view of a strong tradition that views intentions as subject to moral assessment, 21See note 20. we refrain from using the notion, but we do not claim that intention talk makes no sense for non‐human free agents. Note also that while we have approached the subject of free agency via agency attribution, we do not subscribe to the idea that we are here just dealing with a stance we can adopt more or less appropriately, like the so‐called intentional stance. 22See, e.g., Dennett (1987). Free agency is a real phenomenon, and we are interested in its compatibility with the metaphysical claim that the world is indeterministic.

### 2.2 On the relation of free agency and determinism

Having set the stage at the conceptual level, we now come to the question of how free agency and determinism are related (section 2.2.1). This will allow us to state precisely what the purpose of our paper is (section 2.2.2).

#### 2.2.1 Compatibility and incompatibility claims

There are four different claims of compatibility and incompatibility of free agency and (in)determinism that can be made:

(DetCom) Free agency is compatible with determinism.
(DetInc) Free agency is incompatible with determinism.
(IndCom) Free agency is compatible with indeterminism.
(IndInc) Free agency is incompatible with indeterminism.

These claims are largely independent. Logical consistency dictates only that one not hold both (DetCom) and (DetInc), or both (IndCom) and (IndInc), since these pairs are contradictories. All other combinations of claims are logically consistent. Thus, somebody could argue for both (DetInc) and (IndInc), thereby proving that no matter whether the world is deterministic or indeterministic, there can be no free agency. 23For an argument along those lines, see, e.g., Pereboom (2001). A similar conclusion is suggested by Van Inwagen (2000). With specific reference to moral responsibility, the “impossibilist” case has been made by Strawson (1994). At the other end of the spectrum, one could argue for both (DetCom) and (IndCom), thus showing that there can be free agency no matter whether the world is deterministic or not. 24This is the aim of so‐called agnostic compatibilists, or semi‐compatibilists, such as Fischer and Ravizza (1998). We comment on their specific version of compatibilism briefly in section 3.1 below, arguing that it is based on too limited a view of the possible role of indeterminism for free agency. For critical assessment, see also Wagner (2013). (Note that this position would still leave it open whether there *actually* is free agency, since the claims are only about conceptual possibilities, without paying attention to details of what our world is like and which things it contains.)

The four claims differ in the manner in which they can be established. Claims (DetCom) and (IndCom) are compatibility claims. Such claims can be proved directly by example, that is, by providing an actual or a possible scenario in which both notions are instantiated. Thus, in order to establish claim (DetCom) in this direct fashion, one should describe a way the world could be like such that both determinism holds, and there is free agency. Claim (DetCom) could also be established in a somewhat weaker way, indirectly: If one knows that there *can be* free agency  –  e.g., because one knows that *there is* free agency in our world  –  then one has shown that at least one of the compatibility claims (DetCom) or (IndCom) has to be true. If one now also has established an incompatibility claim, e.g., (IndInc), then one knows (since (IndInc) rules out (IndCom)) that claim (DetCom) has to be true. This indirect method of proof is weaker since it relies on stronger assumptions, viz., that one already knows about the possibility of free agency and that one has established an incompatibility claim. Another indirect route to establish claims (DetCom) or (IndCom) would be to piggy‐back one compatibility claim on the other. Thus, if one has established claim (DetCom), one might venture to establish claim (IndCom) by arguing that certain forms of indeterminism do not threaten the argument for (DetCom), 25The so‐called semicompatibilism of Fischer and Ravizza (1998) employs an argument of that form. See also note 27 below. and if one has established claim (IndCom), one might try to establish claim (DetCom) by replacing the relevant instances of indeterminism by some form of determinism‐based unpredictability. 26While we are not aware of a discussion of this point in the free will literature, we have often been confronted with this point in discussions, e.g., by reference to the notion of deterministic chaos or pseudo‐randomness. There are strong arguments showing that certain classes of chaotic deterministic models behave in a way that is empirically indistinguishable from the behavior of certain classes of indeterministic models (see, e.g., Werndl 2011). For the purpose of this paper we can leave it open whether such arguments can be employed for a piggy‐backing strategy in specific cases. Note that many current technical uses of randomness avoid pseudo‐randomness as unsafe and instead employ physical indeterminism. See also notes 30 and 44 below.


Claims (DetInc) and (IndInc) are incompatibility claims. In order to establish such a claim, one has to show that it is impossible that both notions could be true of our world. For example, claim (DetInc) could be proved by showing how one can derive a contradiction from the assumption of determinism together with the assumption of free agency. Such claims can be expected to be harder to establish than compatibility claims: for compatibility, it suffices to give a single example; for incompatibility, one has to rule out a whole range of possible examples.

Note that the dialectical situation we have sketched so far is completely symmetrical between the notions of determinism and indeterminism. For both metaphysical positions, there is a compatibility claim and an incompatibility claim, and the methods for establishing such claims are exactly parallel. The only further remark we can make at this point is that prima facie, it should be easier to establish claim (IndCom) than claim (DetCom). In order to establish claim (DetCom), one has to present a scenario in which both determinism and free agency are exemplified, and since determinism is an extraordinarily strong constraint on ways the world could be like, there are few candidate scenarios. Indeterminism, on the other hand, is defined purely negatively and can be true in various different ways. Thus, there is more room for relevant possible scenarios exhibiting both indeterminism and free agency, which would establish claim (IndCom). 27In fact, the following cheapshot strategy suggests itself: If claim (DetCom) could be established via some deterministic model that involves agency on some planet *A*, one might add some indeterministic happenings in a distant galaxy. The resulting model would then no longer be universally deterministic, but indeterministic. Still, it would continue to exhibit agency on planet *A*. Similarly, it should prima facie be easier, for the same reason, to establish claim (DetInc) than claim (IndInc). 28In fact, with a view to the cheapshot strategy mentioned in note 27, it seems nearly impossible to establish claim (IndInc) without thereby also establishing claim (DetInc). That is, those who hold on to the possibility of free agency can hardly hope to establish the impossiblity of free agency under indeterminism (claim (IndInc)), at least without distorting the claim of universal determinism. On the other hand, it might be possible to establish claim (DetInc) without thereby having to deny the possibility of free agency completely. We stress again that there are many ways for indeterminism to be true. But these are prima facie considerations, which can of course be overruled.

#### 2.2.2 The precise aim of our paper

Given the layout of the dialectical landscape just described, we can now say precisely what the aim of our paper is: We aim to establish claim (IndCom) of the compatibility of indeterminism and free agency, via a direct route: We will provide a possible scenario in which both free agency and indeterminism can be exemplified. (Actually, we provide more than is strictly required for the compatibility claim: We do not just exhibit one instance, but a generic possibility via a whole class of models.) In this paper we do not take a stance on the compatibility question for determinism. 29To indicate where our sympathies lie: We are quite convinced that the incompatibility claim (DetInc) can be established. Good arguments in favor of the claim include the consequence argument given by Van Inwagen (1983), which has sparked a literature of its own, and Kane's argument in terms of ultimate responsibility (for a reappraisal and defence, see Kane 2014). And we make no assumptions about the actual truth of determinism or indeterminism either. 30The truth or falsity of determinism as a metaphysical thesis is certainly a contentious issue in philosophy. A straightforward empirical resolution of the matter seems impossible on conceptual grounds: Indeterminism means the existence of more than one possibility for the future to turn out, but looking back, one always finds just a single realized possibility. Along these lines one can claim that there is no, and in fact cannot be any, empirical confirmation of indeterminism: unrealized possibilities are just that, unrealized, and therefore empirically inaccessible. This would mean that the metaphysical question has to be left open. Any argument in favor of indeterminism (or of determinism, for that matter) therefore has to make extra assumptions, and any such assumption will turn out to be controversial. See, e.g., Wüthrich (2011) for a pertinent discussion. There is not even a firm consensus about the status of specific physical theories with respect to their determinism or indeterminism. Earman (1986, 2006) has shown that the issues involved are extremely intricate. With respect to quantum mechanics, which is certainly a good candidate for an indeterministic theory, philosophical discussions often point out that there are rival deterministic and indeterministic interpretations, with Bohmian mechanics often named as the best contender for a deterministic and realist ontology.It seems to us, however, that the abstract philosophical discussion, especially in relation to quantum mechanics, misses an important aspect of current scientific practice. Hacking (1990) has shown how indeterminism started to be acknowledged as an explanatory resource in the social, and later on also in the natural, sciences, starting in the late 19th century. Meanwhile, indeterminism has become a crucial *technological* resource for our current society. A growing industry centers on providing good sources of randomness for various applications including secure communication, legal contracts, and simulations. There is universal agreement that the gold standard for certified randomness is so‐called device‐independent randomness, which is directly tied to the assumption of the fundamentality of quantum‐mechanical indeterminism. See Acín and Masanes (2016) for an overview, and see note 44 below for a reference to a prominent current implementation effort. The reasoning behind these massive efforts is that only indeterminism at the ontological level can secure true randomness. Before this background, we hold that an interesting critique of the assumption of indeterminism should take the form of an attack on the proposed schemes of certifying randomness, rather than a reference to skeptical ‘underlying determinism’ scenarios. Our point is not that we know that such attacks are impossible, but just that critics of indeterminism seem not to be trying. In a similar vein, Gisin (2018, §8) challenges the Bohmian program to provide some ‘brave new ideas’ that so far seem to be lacking. Physically problematic aspects of the Bohmian program are pointed out by Kiukas and Werner (2010).


### 2.3 On terminology and the space of options

#### 2.3.1 Terminology

Given that the dialectical situation is tidy and symmetrical, it is unfortunate that the terminology that has been established, especially in the recent free will debate, suggests asymmetries between the compatibility claims (DetCom) and (IndCom). An asymmetric treatment has become well entrenched in the debate, but as we will show, the asymmetries are spurious and in fact distort the debate.

Consider the labels used for major positions in the free will debate. 31Apart from Steward (2012), there is not much discussion of compatibility and incompatibility questions about free agency in the action theoretic literature. Steward employs the free will terminology, e.g., in labeling her own position as “Agency Incompatibilism” (Steward 2012, 13). Since the compatibility issues discussed in the free will debate are structurally exactly analogous to the issues we are facing here (the possibility of a realistic reading of a specific type of attribution given a certain metaphysical background), we also employ the free will terminology in what follows. While this has the disadvantage of perhaps triggering too many associations to the free will discussion itself, making up a new terminology would also come at a considerable price and is, in our view, better avoided. First, it is striking that the label ‘compatibilism’ is reserved for claim (DetCom) of the compatibility of free agency and determinism, but that there is no similar label for claim (IndCom). 32Belnap et al. (2001, 204) note that it would be better if claim (IndCom) were called ‘compatibilism’: It is the more important compatibility claim, as it pertains to our arguably indeterministic world. We agree that there should be a separate label for claim (IndCom), and that ‘compatibilism’ would be an apt label, but we will not attempt to change the long‐established terminology here. (Neither do Belnap et al.) Similarly, ‘incompatibilism’ is reserved for claim (DetInc) of the incompatibility of free agency and determinism, 33As we said, Steward (2012, 13) labels her own position as ‘Agency Incompatibilism’ in order to stress that her view targets not free will, but free agency as its precondition. but there is no established label for the incompatibility claim (IndInc). 34While there are no established labels for claims (IndCom) or (IndInc) themselves, there is a commonly used label for a class of arguments that many philosophers hold to establish claim (IndInc), viz., ‘the luck objection’. We will briefly discuss one form of that objection in §4.3. As a symptom of the fact that the debate fails to reflect the symmetry of the positions, one can note that many systematic overviews of possible positions in the free will debate, such as Fischer et al. (2007), are structured around the compatibility or incompatibility of various claims with determinism only. Given such an approach, the possible positions with respect to free agency can be given as a 2 × 2 matrix as shown in Table 1.

**Table 1:** Compatibilism vs. determinism.

	determinism	determinism
	is true	is false
compatibilism (DetCom)	soft determinism	agnosticism (?)
incompatibilism (DetInc)	hard determinism	libertarianism (?)

This table reflects the traditional, century‐old concern that determinism – whether motivated scientifically, theologically, or otherwise – might pose a threat for our freedom. Assuming the truth of determinism, there are, accordingly, specific labels for the compatibilist and incompatibilist positions, viz., ‘soft determinism’ for the combination of the compatibility claim (DetCom) and determinism, and ‘hard determinism’ for the combination of the incompatibility claim (DetInc) and determinism, which rules out free agency. In case determinism is false, however, the opposition between claims (DetCom) and (DetInc) is not really relevant. In the table, we have given only tentative labels for compatibilism/incompatibilism under indeterminism, since the table does not depict the relevant details with respect to claims (IndCom) vs. (IndInc). An agnostic position might be based on the deterministic compatibilist claim (DetCom) and take no stance on the indeterministic compatibility question (IndCom) vs. (IndInc), thus leaving it open whether there can be free agency if determinism is false. The libertarian position, on the other hand, which is often treated as the main (or even sole) rival position to compatibilism for friends of freedom, is defined as a combination of incompatibilism (DetInc), the indeterministic compatibility claim (IndCom), and the actual assumption of free agency and indeterminism – where the latter is often assumed to be established via an indirect argument resting on the assumption of free agency in our world. 35See Wiggins (1973). Note that Kane (1998, 13) phrases his ‘intelligibility question’ in the following way: “Can we make sense of a freedom or free will that is incompatible with determinism?” This question combines two issues that we wish to keep separate: the incompatibility claim (DetInc) and (reading ‘make sense of’ as ‘establish the possibility of’) our claim (IndCom).


In our view, libertarianism is not a helpful alternative to compatibilism because it is too specific, combining three (or even four) logically independent claims under one label. Of course, such a package deal is dialectically more fragile than compatibilism, since it can be attacked in many different ways, especially since it spans both action theory (in view of the compatibility and incompatibility claims involved) and metaphysics (in view of the fact that libertarians assume the truth of indeterminism). As we said, we do not address the metaphysical issue at all in this paper. And with respect to compatibility and incompatibility claims, our task here is just to defend one aspect of libertarianism, viz., claim (IndCom) of the compatibility of free agency and indeterminism. This claim is not a rival to the compatibilist claim (DetCom) at all. 36In fact, given how strong a metaphysical assumption determinism is, claim (DetCom) almost implies claim (IndCom) – but not in an interesting way (see note 27). Our model is meant to establish claim (IndCom) in an interesting, non‐derivative way. And just as defenders of (DetCom) can remain silent on the incompatibility question (IndInc), so we can remain silent on the incompatibility question (DetInc) in establishing (IndCom). 37Thanks to Verena Wagner for discussion in connection with her paper “Reconciling projects”, presented at Konstanz on March 20, 2015.


#### 2.3.2 The space of options

With respect to the actual space of options in the discussion of free agency and determinism, a useful, symmetrical 2 × 2 table can picture the possible positions w.r.t. (DetCom)–(IndInc) as shown in Table 2.

**Table 2:** Compatibility and incompatibility claims.

compatible with free agency	indeterminism: yes (IndCom)	indeterminism: no (IndInc)
determinism: yes (DetCom)	(A) freedom possible anyway	(B) freedom only under det.
determinism: no (DetInc)	(C) freedom only under indet.	(D) no freedom

This table shows that, as we remarked above, a position that falls under the traditional label of compatibilism is not yet fully detailed: Compatibilism (DetCom) stands for the upper row of the table, which comprises two fully specified positions, (A) and (B). Of these, position (B) is the position advocated in the title of a paper that puts forward what has become known as the “*Mind* argument” 38That name is due to the fact that the paper, and other papers with similar claims, appeared in the journal, *Mind*, in the 1930s. See Van Inwagen (1983, 16) and Franklin (2011) for the terminology. (Hobart 1934): “Free will as involving determination and inconceivable without”. Not all compatibilists nowadays want to hold on to such a position, since they may not wish to make the possibility of freedom dependent on the thesis of determinism. (Most authors assume that science, not philosophy should give the final verdict on the truth or falsity of determinism.) Many compatibilist authors nowadays want to remain neutral with respect to the truth or falsity of determinism. 39For example, writing about free will rather than free agency, Fischer (2012, 4) stresses that moral responsibility should not be “hanging on a thread” in the sense of depending on “subtle ruminations of theoretical physicists”. In that case, however, they will have to argue for claim (IndCom) in order to avoid commitment to position (B). 40The fact that freedom‐affirming compatibilists who want to avoid a commitment to determinism have to tackle claims (IndCom) and (IndInc) is also noted by Franklin (2011, 202n5). Such agnostic compatibilists are thus also facing the task to which this paper is devoted. They should therefore welcome the model we are proposing in this paper. At any rate, the reality of agency as well as the truth or falsity of determinism should be discussed independently of the conceptual compatibility claims here at issue.

## 3 A model for free agency under indeterminism

Our task in this section is to motivate (section 3.1) and then explain (section 3.2) a model of free agency under indeterminism, which is based on the recent AI learning and deliberation framework of *projective simulation* (Briegel and De las Cuevas 2012). In section 3.3 we argue that the model can indeed exemplify free agency under indeterminism. We provide a discussion of a concrete example in section 4.

### 3.1 Motivating the model

In our constructive task of establishing the compatibility claim (IndCom), we want to show how indeterministic randomness can be a useful and in fact constitutive resource for a free agent. 41We stress again that our task of establishing claim (IndCom) is logically independent of taking a stance on claims (DetCom) vs. (DetInc). In the model we will propose, indeterministic randomness plays a constitutive role. By proposing this model we do not, however, claim that there could not be a different model for free agency that could be realized in a deterministic world. While we doubt that this is possible – see note 29 – we do not address the issue of the compatibility of free agency and determinism in this paper at all.


Here is a first way in which randomness can be useful for an agent. The model to be described below goes far beyond this, but we offer the following considerations as a first step toward the idea that as an agent, randomness can be your friend. Consider tie‐breaking: An agent is in a situation in which a particular choice has to be made, but the actual choice does not matter. Famously, this is the situation of Buridan's ass situated symmetrically between two equally attractive stacks of hay. Since there is nothing in the situation to tip the balance one way or the other, the creature has to find a way to break the tie if it wants to avoid starvation. Randomness can come in handy here: It is perfectly appropriate to tie the tie‐breaking to some random event. And this is so independently of whether the randomness can be traced back to some microscopic happening in the agent's brain, or whether it is enforced by tossing a coin (or, better, sending a photon through a beam splitter). So, given that agents from time to time are facing choices whose outcome does not matter, having a built‐in random tie‐breaking mechanism would be helpful. At this point, however, the randomness of the tie‐breaking seems to play no crucial role; it is just one option for breaking ties, and there is no strong incentive for the agent to employ randomness. We will not take a stance on whether such tie‐breaking situations, in which there is really *nothing* in the situation to tip the balance, are common for us humans or not; they may in fact be rare. 42Ironically, it seems that among the best candidates for mere tie‐breaking situations we find the experimental settings of neuroscientific free‐will experiments such as Soon et al. (2008). In that experiment, participants have to choose, roughly equally, between the two options of clicking left or right. Whether you click left or right in such an experiment really does not matter at all.


Here is a next kind of situation in which randomness can really help an agent in a specific and crucial way. Animals trying to escape from a predator will be caught easily if their escape behavior is predictable. So it has great survival value for typical prey to show erratic, unpredictable escape behavior. And such behavior is found in many experimental studies, e.g., in cockroaches and in flies. 43See, e.g., Couzin and Krause (2003), Domenici et al. (2008; 2011), and Brembs (2011). Here the task is not just tie‐breaking (the animal has to run away in *some* direction rather than stay where it is), but tie‐breaking *in an unpredictable way* (the predator should not be able to guess which direction and which path the animal will choose). While unpredictability may be realized in different ways, if physical randomness is available, it could be an obvious and biologically natural means to achieve unpredictability, so that here we have a strong case for a positive role of randomness in agency.

Still, these tie‐breaking examples only show that randomness can be useful for an agent in the sense that successful agents will profit from having sources of randomness available to handle special types of situations. 44Tie‐breaking may arguably also be invoked when the agent's choice does matter, but the agent's preferences are incomparable rather than tied. And at a much higher level of sophistication, randomness is required for us human beings as users of modern communication devices as well: The security of communication channels, including, e.g., internet connections, depends on the availability of genuine randomness for encryption (Shannon 1949). Initiatives such as the US NIST beacon project work toward providing certified and publicly documented genuine randomness for a variety of technological and commercial purposes. See https://beacon.nist.gov for the US service. A model for free agency under indeterminism should provide more if it is to make a positive contribution in the current debate about free agency: Such a model should not stop at making the general point that randomness can be a useful resource, but show explicitly how randomness can be a useful element in the dynamical coming to be of an individual, specific action.

At this point, two approaches are available. The common approach is to start with a basically deterministic model of agency, such as offered, e.g., by various compatibilist analyses of agency, and add a random element at the right place of the causal history. 45All the options for indeterministic theories of agency discussed in the overview by Franklin (2011), e.g., the so‐called ‘deliberative libertarian’ theories of Mele (1999) and of Clarke (2000), are of that type. This, in turn, could take the form of showing how such a random element would be tolerable, i.e., it wouldn’t destroy the basically deterministic model, 46See, e.g., Fischer and Ravizza (1998, 253). or it could take the form of showing how such a random element provides a positive contribution to the given, otherwise deterministic agency model. 47See, e.g., Kane (1998, ch. 5) on ‘self‐forming actions’ as indeterministic happenings. See the essays in Palmer (2014) for discussion.


The other approach, which we call *deeply stochastic agency modeling*, is not to start with a deterministic model at all, but to work out a stochastic model, in which indeterminism is the central resource for the model's dynamics. Stochastic models are well known in the sciences. Important examples include Einstein's explanation of Brownian motion (Einstein 1905) and Fisher's model of natural selection in population genetics (Fisher 1930). Such models have a broad range of applications both in the natural and social sciences (Van Kampen 2007; Gardiner 2009). In what follows, we propose such a stochastic model: free agency that is based on a fundamentally random process. 48For the model at hand, there is a specific additional reason, over and above what we said in note 26, why a deterministic (pseudorandom‐based) substitute would not be of much use: Such a variant would preclude straightforward quantum extensions of the model, which are currently being studied in physics and in AI (Paparo et al. 2014; Dunjko et al. 2016). According to that model, a learning agent comes to act at the end of an underlying stochastic process in her memory that is extended in time. Randomness thereby plays a constitutive role: Options and considerations unfold stochastically and culminate in action.

### 3.2 The model: Projective simulation

We now describe a stochastic process model of free agency under indeterminism based on an associative memory organization and random option selection. The model, called *projective simulation* (PS), is built around a dynamic network of episodic memory (Briegel and De las Cuevas 2012). 49Briegel and Müller (2015) use the model in an action‐theoretic context, but do not relate their discussion to the options that are open in the freedom debate. Stochastic transitions in such a network, suitably interpreted as an agent's considerations, are the basis of an explicit, formally well‐specified model of deliberation in a learning context.

**Figure 1:**
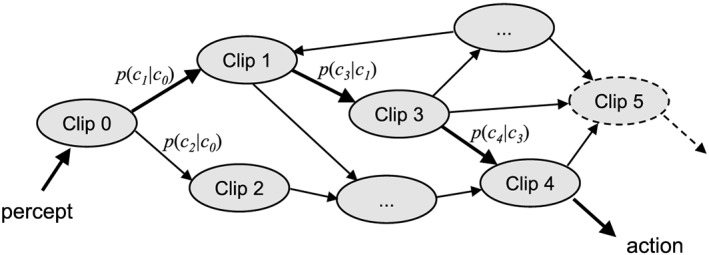
Episodic and compositional memory as a stochastic network of clips (Figure adapted from Briegel and De las Cuevas 2012).

The basic memory structure of an agent embodying the projective simulation model is *episodic and compositional memory* (ECM), which is constituted by a dynamic network of so‐called *clips*. Clips are the units of episodic memory and correspond, in the simplest case, to memorized actions or percepts, or short sequences thereof. Such a network is depicted in Figure 1. The network is dynamic both with respect to its topology (number of clips and connections between them) as well as with respect to the weights of the connections between adjacent clips, which change through learning.

The basic process of deliberation for a PS agent can be expressed within the percept‐action framework of artificial agents (Russell and Norvig 2011) and goes as follows. Given a specific state (topology and weights structure) of the ECM and triggered by some perceptual input, a first memory clip is activated. Subsequently, a random walk through the clip network ensues, involving a number of transitions that is not determined beforehand, until activation is coupled out, triggering some motor action. The random walk through the clip network follows the weights (probabilities) as specified in the given state of the ECM. In Figure 1, the bold arrows indicate such a random walk from a percept to an action consisting of five steps. The arrows are labeled by the respective probabilities; for example, *p*(*c*_1_| *c*_0_) is the probability that, given that Clip 0 is activated, the next activated clip is Clip 1. These probabilities, or weights, are built up through the agent's learning history and thus encode her past experience connecting sensory input to action output, including the consequences (good or bad) that these actions had for the agent. Weights are updated after an action has been performed and feedback has been received (as part of the perceptual input), e.g., by strengthening those connections that were activated during the random walk leading from percept to rewarded action. The updating of the ECM may also include the stochastic generation of new clips through variation or composition of existing ones, which changes the topology and size of the associative network. This provides additional flexibility for the agent to develop different patterns of deliberation and action over time.

In one run of projective simulation, a given percept as input at time *t*_0_ can lead to any of a number of actions as outputs, and output can be triggered at different times *t*_0_ + *m*Δ*t* corresponding to a deliberation length of *m* transitions (each taking the time Δ*t*, assuming a simple model with uniform dynamics). Various refinements and extensions of the model are possible and have been explored. These include different learning schemes, e.g., with the capacity to generalize (Melnikov et al. 2017), or with meta‐learning, which involves the adaptation of the learning parameters themselves (Makmal et al. 2016). Further extensions concern the possibility of quantizing the model, which allows for a quantitative speed‐up in deliberation and active learning (Paparo et al. 2014; Dunjko et al. 2016). Here we will focus our discussion on the basic structure in order to make our case as transparently as possible. It should be noted up front, though, that lack of complexity of the model is due to our discussion of the simplest base case, and that more complex schemes are readily available. 50Recent developments include applications in robotics (Hangl et al. 2016, 2017a,b) and in the design of novel quantum experiments (Melnikov et al. 2018).


### 3.3 Projective simulation: Free agency under indeterminism

Here is how the projective simulation model provides the ingredients for free agency, and thereby witnesses our claim of the compatibility of free agency and indeterminism.

First, it is clear that the model is indeterministic – it is based on an indeterministic random walk for each coupling of sensory input (percept) to motor output (action). It should also be noted that this indeterministic aspect is a fundamental feature of the model in the same way in which other well‐known models in physics, such as the ones referred to at the end of section 3.1, are fundamentally indeterministic. In projective simulation, the indeterminism is not added on top of an otherwise deterministic model, and so the model cannot be adequately described as a randomization of some other, deterministic, deliberation scheme. Therefore the model truly exemplifies the less explored strategy of deeply stochastic agency modeling described at the end of section 3.1.

Second, we claim that the model provides all the ingredients needed to exhibit free agency. Together with the previous step, this shows that the model is a witness for our compatibility claim.

In the description we give in the following, we adopt a certain semantics for the clips in the agent's ECM network: we will speak about the content of the clips as *considerations*. This is meant to provide the basis for associations, and thus to make sense of the stochastic hopping of activation in the network, including the coupling in of percepts and the coupling out of actions. At the same time, the terminology of ‘considerations’ is meant to be low‐level enough not to suggest that the agent somehow interprets the content of her memory clips consciously, or reflects on them.

We proceed by establishing that the model meets the criteria for free agency laid out in section 2.1.2. Thus, we have to argue that the actions of a PS agent can be causally relevant, non‐rigid in a flexible and adaptive way, and sensible. *Causal relevance* is clear since a run of projective simulation is itself a causal process that terminates in some action. The *non‐rigidity* of actions is also obvious: Given one and the same perceptual input and the same internal state, several actions are possible – the agent's behavior is not a hard‐coded reflex. Over a short time‐scale, that non‐rigidity is also *flexible* and not erratic: the agent goes through a process of activating different considerations before triggering an action. On the time‐scale of a single action, the deliberation process – the transition from perceptual input to resulting action – is guided by the current structure of the agent's memory (see section 4 for an explicit example). On the longer time‐scale of the agent's development, the agent's behavior is also non‐rigid in the sense of being *adaptive*. The agent learns from past experiences by adjusting its structure of considerations, e.g., by reinforcing connections that have led to success (perceived positive feedback), and by creating new clips and making them available for long‐term adaptation as well. Thereby it fits its behavior to the environment it finds itself in. In summary, the PS agent fulfills all the necessary conditions for free agency formulated in section 2.1.2: causal relevance, non‐rigidity, and flexibility and adaptivity.

In section 2.1.2, we spelled out a notion of sensibility as part of a sufficient criterion for free agency. We argued that an agent fulfilling the necessary criteria that we just discussed is a free agent if it acts on its own considerations, and develops the structure of its considerations in a meaningful way over time. The issue is, therefore, whether the PS agent's actions are really *sensible* in this sense.

In favor of our claim that a PS agent's actions are sensible, we can offer two main arguments that relate to the mentioned two different time‐scales. First, while the projective simulation process is indeterministic, each single instance of that process makes sense before the background of the agent's learning history reflected in the transition weights and, more generally, in its memory structure at the given time. On the short time‐scale of an individual action, a single stochastic process leading from sensory input to that action represents the dynamical succession of considerations in the agent's associative memory structure. Each clip in the ECM network that is activated in the course of that deliberation process represents some consideration that is relevant for the situation at issue, and the actual succession of these clips represents the associative progression of considerations leading to the agent's action. While this individual process is stochastic, i.e., its concrete course is not fixed beforehand, the process is clearly specifically the agent's: Both the topology of the ECM network and the clip‐to‐clip transition probabilities are shaped by the agent's actual past and learning history, marking the practical deliberation process as belonging to that individual agent.

Second, on the longer time‐scale of learning and forming behavioral dispositions, each individual deliberation‐action process makes sense because it contributes to the agent's individual history and to its development as an agent. Each such process allows for learning through the feedback (in the simplest reinforcement learning scheme, a perceived reward) that the agent receives from the environment. That feedback first of all affects the transition weights between clips that were activated during the actual deliberation process. Furthermore, as part of the projective simulation scheme, new clips may be created out of already existing ones, which need not correspond to any factual experience in the agent's past. If the activation of such ‘fictitious’ clips, as part of a random walk, leads to rewarded actions, their embedding into the clip network will be strengthened and they may become an integral part of the episodic clip network. This process of random clip creation, together with the mentioned dynamics, can then lead to new options for action, as well as to new paths of considerations in the agent's memory.

Also related to the longer time‐scale of learning, we can note that based on indeterministic decision processes with learning through feedback, a PS agent can develop (almost) deterministic reactions to specific stimuli. PS agents thus need not be unreliable or haphazard. In some cases they can exhibit behavior typical of hard‐coded routines (even starting from quite arbitrary connection weights in their memory): given proper reinforcement, an agent can, as it were, learn to become a rigorist about certain forms of behavior in specific circumstances. This allows the model to capture in a sensible way the dynamics of building up strong habits, or a firm character, as one might say, in the face of almost unlimited options for action. 51In the free will debate, indeterminism‐based accounts (i.e., libertarian theories) are often confronted with a ‘challenge from luck’, one aspect of which is exactly how to account for a firm moral character in the face of indeterminism (see also section 4.3). Learning through feedback, in our view, provides an adequate answer: Over time, an agent can acquire firm responses to certain types of situations, while still remaining somewhat flexible in most cases. In a similar vein, although limiting the role of indeterminism to torn decisions leading to what he calls “self‐forming actions”, Kane (1998) stresses the role of an agent's history in accounting for moral responsibility. Rigorous behavior is, however, not always warranted, and the model does not enforce it. In some cases, such as in tie‐breaking, a PS agent may still react completely randomly, and in other cases in which different considerations favor different actions, it will show the appropriate flexibility in its actions. So, a PS agent can learn to behave reliably when this is required, and still retain a certain amount of flexibility, up to complete randomness, when that is more appropriate. The PS agent's adaptive use of indeterminism makes good sense.

Within the PS model, therefore, we use indeterminism as a central and basic resource. Importantly, this does not mean that we expose an otherwise deterministic agent to certain random processes. The PS model is not based on an underlying deterministic deliberating agent that is somehow ‘randomized’ to arrive at the model in a second step. The random processes we are referring to here are not something external that would randomize the agent's actions (as if the agent was given independently and beforehand, and then perhaps enslaved by outside randomness). On the contrary, the random processes form a constitutive element of the agent's memory and the very process of decision finding.

It should be pointed out that the model of projective simulation, including its rules for transitions and compositions in clip space, represents a specific model of reinforcement learning in a physically inspired approach to (quantum) artificial intelligence. It is meant to be a simple model for natural and artificial agents that can learn and show flexible and sensible behavior. We do not claim to give an account of any deeper or more advanced aspects of human agency such as free will. For our purposes, projective simulation serves as a formal model of free agency, where the process of decision finding that precedes an action in a given learning environment can be mapped out in detail.

## 4 The dynamics of projective simulation: An example

Our description of the PS model and of its interpretation so far has been fairly abstract. In order to balance our description, we now give an (oversimplified) example of the dynamics of projective simulation in which the abstract talk of considerations is tied to a concrete situation. Here is our little story: We assume that while you are typing at your sunny desk, your cat is sitting on the floor and is about to either jump on the desk, perhaps to lie down on your keyboard, or to walk away. We will use this example both to illustrate the PS dynamics and to strengthen our argument for the possibility of attributable free agency under indeterminsm.

### 4.1 A toy model

**Figure 2:**
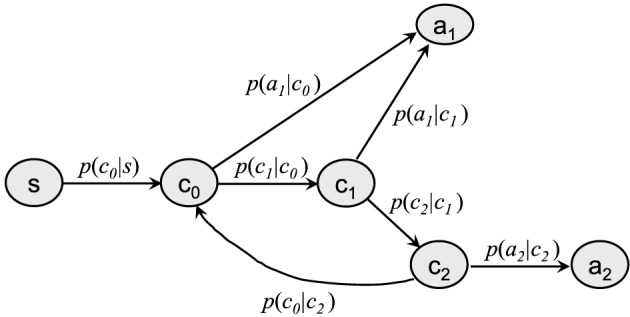
The clip network of our toy model. Arrows are labeled by the respective transition probabilities.

In order to keep the discussion managable, we limit ourselves to the toy model shown in Figure 2, which comprises just one possible input percept *s*, two possible actions *a*_1_ and *a*_2_, and three intermediate considerations *c*_0_, *c*_1_, and *c*_2_.

We interpret these clips in the cat's memory, with reference to our little story, as follows: Percept *s* represents the situation that the cat is in: sitting on the floor, sunlight on the desk, her owner sitting at the desk typing. Action *a*_1_ represents jumping, action *a*_2_, walking away. The given percept *s* triggers consideration *c*_0_ with probability *p*(*c*_0_| *s*) = 1. The associative considerations linking percept *s* to one of the actions *a*_1_ or *a*_2_ in the stochastic ECM network are interpreted as follows. Consideration *c*_0_ represents remembered episodes of the cozy feeling of the warmth of a sunny patch on the desk. This consideration can, in the given network, either trigger action *a*_1_ (jumping on the desk), with probability *p*(*a*_1_| *c*_0_), or a transition to clip *c*_1_, with respective probability *p*(*c*_1_| *c*_0_) = 1 − *p*(*a*_1_| *c*_0_). Consideration *c*_1_ represents remembered episodes of the cat's owner giving attention to her when she had disturbed him while he was typing. Again, this consideration can, in the given network, either trigger action *a*_1_ (jumping on the desk), with probability *p*(*a*_1_| *c*_1_), or a transition to clip *c*_2_, with respective probability *p*(*c*_2_| *c*_1_) = 1 − *p*(*a*_1_| *c*_1_). Finally, consideration *c*_2_ represents remembered episodes of the owner brushing the cat quickly off the desk after she had jumped there. The two possible outcomes after this consideration in the given network are to trigger action *a*_2_ (walking away), with probability *p*(*a*_2_| *c*_2_), or to transition back to clip *c*_0_ (which we can interpret as reconsidering the situation), with respective probability *p*(*c*_0_| *c*_2_) = 1 − *p*(*a*_2_| *c*_2_).

### 4.2 Calculating the dynamics

Based on this network structure and given specific values for all the mentioned transition probabilities, we can describe the fine structure of the cat's possible courses of deliberation and action quantitatively. In order to simplify the math, let us assume that all transitions from a given node have equal weight, such that, for example, both possible transitions from clip *c*_1_ – jumping (*a*_1_) or arriving at the next consideration *c*_2_ – have equal probability of $p\left(a_{1}|c_{1}\right)=p\left(c_{2}|c_{1}\right)=\frac{1}{2}$. An agent who has learned from experience will of course have adjusted the transition weights. For example, if the cat has often been rewarded for jumping on the desk, her weight *p*(*a*_1_| *c*_1_) for the *c*_1_ → *a*_1_ transition (jumping on the desk) will be much larger than the weight for the transition *c*_1_ → *c*_2_ (recalling being brushed off the desk).

The shortest path through the network leading from percept *s* to some action is the following:

$$s\to c_{0}\to a_{1}.$$

The probability of that path, given *s*, is $1\cdot \frac{1}{2}=\frac{1}{2}$. In Table 3, we list a few more paths with their respective probabilities. (Note that due to the cyclic structure of the model network – the possible transition from *c*_2_ to *c*_0_ enables arbitrarily long cycles – there is no limit to the length of possible paths, but of course the probability of paths becomes smaller the longer they are.)

**Table 3:** Possible paths in the network of Figure 2.

Path	length *l*	probability *pr(l)*	jump or walk away?
*s* → *c*_0_ → *a*_1_	2	0.5	jump
*s* → *c*_0_ → *c*_1_ → *a*_1_	3	0.25	jump
*s* → *c*_0_ → *c*_1_ → *c*_2_ → *a*_2_	4	0.125	walk away
*s* → *c*_0_ → *c*_1_ → *c*_2_ → *c*_0_ → *a*_1_	5	0.0625	jump
*s* → *c*_0_ → *c*_1_ → *c*_2_ → *c*_0_ → *c*_1_ → *a*_1_	6	0.03125	jump
*s* → *c*_0_ → *c*_1_ → *c*_2_ → *c*_0_ → *c*_1_ → *c*_2_ → *a*_2_	7	0.015625	walk away
…	…	…	…

Given our simple stochastic dynamics, the total probability that the cat's deliberation will finally lead to jumping at some time after the time of the percept *s*, *t*_0_, under the assumed weights, is the sum of the probabilities of all paths leading to jumping, which comes down to *p*_jump_ = 6/7. The probability for walking away is, accordingly, *p*_walk away_ = 1 − *p*_jump_ = 1/7. Note that by adjusting the weights, we can arrange for any ratio of jumping vs. walking away. 52Consider, for example, a network in which the probabilities in all branchings are adjusted in such a way that the direct transitions to *a*_1_ (i.e., those from clips *c*_0_ and *c*_1_) occur with probability *p* and the direct transition to *a*_2_ (i.e. the one from clip *c*_2_) with probability *1‐p* (with 0 ≤ *p* ≤ 1). The effective probability for jumping is then given by the expression $p_{\text{jump}}=\frac{p\left(2,-,p\right)}{1-p{\left(1,-,p\right)}^{2}}$. An effective 50:50 distribution among the two options is obtained for the configuration with *p* ≃ 0.245, which is the real‐valued solution of the cubic equation *p*^3^ − 4*p*^2^ + 5*p* − 1 = 0.


In the given model, jumping can occur immediately, after two steps (each of duration Δ*t*), at time *t*_0_ + 2Δ*t*, but also after a much longer deliberation time, e.g., after 12 steps, corresponding to time *t*_0_ + 12Δ*t*. Similarly, walking away can occur after four, after seven, or after more steps. 53In our simple model, the action that can result after a given number of deliberation steps is uniquely determined. This feature does not hold in general; it is due to the simple structure of the network. In a network with an added transition from *s* to *c*_2_, for example, both walking away and jumping are possible after two steps. To repeat, the example is only meant to illustrate the basic dynamics of the PS model, not to provide a realistic picture of an actual cat's deliberation process in its full complexity.
^,^ 54The average time of deliberation in our simple model, that is the average time after which the cat acts (either jumping or walking away) is

$${\left\langle T\right\rangle }_{\text{delib}}=\sum_{l=2}^{\infty }l\Delta t\cdot \mathrm{pr}\left(l\right)=2\Delta t\cdot 0.5+3\Delta t\cdot 0.25+4\Delta t\cdot 0.125+\ldots =3\Delta t.$$

For those possibilities in which the cat eventually jumps, the average time of deliberation is approximately ⟨*T*⟩_jump_ ≃ 2.76Δ*t*. For those possibilities in which the cat eventually walks away, the average time of deliberation is approximately ⟨*T*⟩_walk away_ ≃ 4.43Δ*t*. This difference illustrates that walking away or jumping, in this model, is not a point‐like event or decision, but an extended process with temporal fine structure.


### 4.3 A comment on the replay argument

With our toy model we have illustrated a simple memory structure of considerations leading from perception to action via different paths. In Table 3 we have shown some of these paths in detail, including values for the paths’ probabilities. These paths provide an internal description of the stochastic development of the agent's considerations. We have also given an external description of which actions the stochastic development leads to (the mere input – output coupling), both for the individual paths differentiated internally (see the right‐hand column in Table 3) and in aggregate fashion (see the values for *p*_jump_ and for *p*_walk away_ given above). Before this detailed background, is it useful to discuss an analogoue of an argument that in the free will debate is often taken to speak against the compatibility of free will and indeterminism: the so‐called luck objection, and specifically, its perhaps strongest form, Van Inwagen's replay argument (Van Inwagen 2000). As we have stressed many times, we are here not concerned with the free will debate, but the replay argument has a structure that can easily be transferred to our discussion of free agency.

The basic worry behind any form of the luck objection is that if something happens indeterministically, it is due to chance, and thus not due to an agent. In this general form, the objection is easily dismissed – it construes chance as an agent herself and then invokes a rivalry between being due to chance and being due to the agent in question. However, calling something ‘due to chance’ is not yet an analysis, but a metaphorical redescription. A lot more has to be said in order to show that the mere occurrence of an indeterministic event in the history of a happening disqualifies that happening from being the action of an agent. A stronger form of the luck objection points out that indeterministic happenings have no explanation. A fortiori, they cannot be explained by the involvement of the agent, and thus, they cannot be attributed to the agent, because attribution implies explanation. This variant of the objection is more serious, but it can also be dismissed once the notion of explanation in question has been clarified. It is true that if at time *t*_0_, it is both really possible that at some later time *t* an event *E* happens, and really possible that *E* fails to happen at *t*, then given that *E* happens, there can be no full contrastive explanation for why *E* happened rather than failed to happen. After all, both outcomes were possible as of *t*_0_, so it is analytic that no full (sufficient) contrastive explanation can be available. On the other hand, it is simply not true that such an event cannot be explained. 55If you think one always needs to have a contrastive explanation for one's choices, consider the following joke: A mother buys her son two ties for his birthday. Next time she sees him he's wearing one of them, so she says to him “What, didn’t you like the other one?” Consider the cat in our little story. If the cat walks away at time *t*_0_ + 4Δ*t*, then the actual path of considerations from percept *s* to action *a*_2_ explains why the cat did that: she did so on the basis of the active consideration *c*_2_ about the remembered negative effects of jumping. If one claims that such an explanation is not enough, one thereby just stipulates that indeterminism precludes explanation and, thus, attribution. This is, however, just an inadequate stipulation that does not match our practices of explanation and attribution. 56See, e.g., Anscombe (1971) for a discussion of the idea of explanation as ‘saying enough’, and Feynman (1965, 147) for the famous example of causal explanation under indeterminism referred to by Anscombe.


Van Inwagen's replay argument is more specific than the two variants of the luck objection just mentioned, and therefore more serious. He considers a real action at some time (think of the cat walking away at time *t*_0_ + 4Δ*t*) and the indeterministic real possibilities at some previous time (think of the cat registering percept *s* at time *t*_0_). Van Inwagen prompts us to imagine the situation to be replayed 100 times from time *t*_0_ on, and then to record the frequencies of the occurrence of the various possible actions. These frequencies can be interpreted as probabilities, and this shows, in his view, that the original action cannot really be attributed to the agent because it was just one run of a chance process, like a coin toss.

With the projective simulation model, we can meet the replay challenge head‐on. 57We repeat that the replay argument is originally given in the context of a discussion of free will. In fact, Van Inwagen's actual example is of a woman who has to make a morally important choice about either lying or telling the truth. The woman, Alice, in fact tells the truth, but according to Van Inwagen, a consideration of possible replays shows that we cannot praise Alice for her actual truth‐telling, given that it is the result of a random process. In this paper we do not discuss free will and moral attribution, just agency attribution. We do not take a stance on whether there might be additional factors in moral scenarios that might make moral attribution under indeterminism more problematic than agency attribution under indeterminism. Certainly moral attribution is a more complex phenomenon than agency attribution, which it presupposes. We believe, however, that our discussion should also be helpful for the free will debate. We just said that the PS agent can be described internally and externally. The internal description in terms of the detailed dynamics of the individually possible paths shows which transitions between memory clips can lead from percept to action, thereby providing an action explanation based on the agent's historically grounded clip network. A concrete path also provides the handle for learning through feedback, e.g., by strengthening those connections that were activated before an action that was rewarded. Thus, such a path also influences the agent's behavioral dispositions. The action is thereby fully embedded in the agent's ongoing developmental history. The internal description provides a point of view from which the stochastic PS dynamics makes sense for the agent.

The replay argument, on the other hand, is phrased in terms of an external description that only considers the final step of the dynamics, which results in the action. This description lacks the detail to make an individual run understandable and thereby attributable. The external description shields the resulting action from the actual dynamics and thereby provides a description that indeed only offers an interpretation of what is going on as a chance process. But for a PS agent, such a description lacks the crucial detail of the internal dynamics. And apart from this structural point, there is also an important quantitave point to be made about the timing of actions. In our description of the internal dynamics in section 4.2, we have seen that the length of a deliberation process in the agent's memory is not fixed beforehand, so that an action can result at different times. The replay argument falsely suggests that the different possible actions – the cat's jumping or walking away, in our toy example – are alternatives for the same time given the same relevant past. The internal description shows that this is doubly wrong. First, there is a difference in the relevant past in our toy model: The different actions are linked to different considerations, and this difference explains how the different actions all make sense. 58This aspect is often neglected. For example, Mele (2006, 58) assumes an agent who “freely decided at *t* to *A*” and an alternative “with the same past until *t*”. Our discussion points out that the same past may lie a little while back. Second, the different actions need not be alternatives for the exact same time. In fact, in the network of Figure 2, if clip *c*_1_ is activated, action *a*_1_ can result immediately, but the immediate alternative to action *a*_1_ is not action *a*_2_ (which would not make sense as flowing from the consideration *c*_1_), but the associative activation of a different consideration, *c*_2_, which can then lead to action *a*_2_ in a sensible way. Thus, the immediate alternative to a certain action is normally not a different action, but rather reconsidering, or continuing to deliberate. 59This point is also suggested, e.g., by Broad (1933, 240), Keil (2007, 115), and Steward (2012, 155ff.). In fact, already in our toy model, one of the alternatives to action *a*_1_ at time *t* is *the same action*, *a*_1_, but *at a later time*, e.g., at *t* + 3Δ*t*. This makes good sense phenomenologically: Often it takes a while to get oneself to do something, and while one is deciding, different considerations become salient, mostly with an associated next action. What one does is based on the consideration that was active immediately before one acts, and it is not determined beforehand how long one will deliberate. So even in our toy model with just two physical actions *a*_1_ and *a*_2_, the internal dynamics forces one to acknowledge a further possibility, viz., continuing the process of deliberation. 60Note that the indeterministic process of either continuing to deliberate or to act immediately that we refer to here when describing the internal dynamics of a PS agent *is not itself* an action, but just a part of the random process that realizes an action in the PS model. Phenomenologically, we know that sometimes we consciously decide to continue deliberating. This phenomenon is different, because it *is itself* an action. For example, one may feel drawn to send off an angry text message, but actively force oneself to reconsider, having learned from bad past experiences. Such active reconsidering occurs as a separate action, and therefore on a higher level than the underlying PS dynamics.


A longer‐term perspective further strengthens the case for the adequacy of the internal perspective. The agent's past is what shapes the transition weights that form the background for the stochastic ECM dynamics at a given time. Feedback after an action can result in changes in these weights, thereby influencing future behavior. Such a longer‐term perspective is typically missing in discussions of the luck objection. The longer‐term perspective is present in Kane's model of self‐forming actions, which, while targeting free will, bears some relevant similarity to our account (see note 51). But it is completely absent from Van Inwagen's argument.

Note that even if the agent's memory structure is such that the actions *a*_1_ and *a*_2_ in the end both occur with a probability of $\frac{1}{2}$, 61See note 52 for the respective transition probabilities in our toy model of Figure 2. it is not adequate to think of the agent as tossing a fair coin to decide what to do. The indeterministic decision process does not just provide an outcome satisfying certain statistics, but in each and every run provides a path of considerations that is shaped by the agent's past experience. A coin toss would not do that; it would destroy the sensibility of the agent's action. And it would also not provide a foothold for the longer‐term dynamics of learning that free agents can undergo. If in the case of a meaningful decision, the link between action and considerations is given up, this threatens the agent's integrity. The mere fact that a deliberation process is indeterministic, on the other hand, does not.

## 5 Conclusion: A model‐based argument for the compatibility of free agency and indeterminism

In this paper, we have proposed a novel approach to the question whether there can be free agency under indeterminism. That question is of crucial importance for our practical self‐conception and for the freedom debate: There are good reasons to assume that our world is indeterministic, and we consider ourselves to be free agents in that world, so we should understand in which way our freedom could be compatible with indeterminism. That question is surprisingly little discussed.

The novelty of our approach lies in the aim we set ourselves and in the chosen means. Our aim was to argue only for a positive compatibility claim: for the compatibility of free agency and indeterminism. In the free will debate, the only compatibility question that is regularly discussed as an isolated question is about the compatibility of freedom and determinism. The compatibility of free agency and indeterminism, however, is just one item on the to‐do list of would‐be agency libertarians, i.e., of those who hold a complex package deal of the incompatibility of free agency and determinism, the compatibility of free agency and indeterminism, the existence of free agency, and the truth of indeterminism. As we pointed out, this setting up of the debate gives a biased picture of the available positions. Our aim is much more modest than that of establishing agency libertarianism. We need not be concerned with the compatibility or incompatibility of free agency and determinism, nor with the actual truth of indeterminism. All we were after in this paper was to prove the compatibility of free agency and indeterminism, i.e., the possibility of a world that features both free agency and indeterminism.

With respect to means, we opted for the direct route to establishing our compatibility claim via an explicit, mathematically well‐defined and physically motivated class of models. We described the agency model of projective simulation and argued that it exhibits the sought‐for combination of free agency and indeterminism. Thus our main argument is a constructive one, exhibiting examples of what we claim to be possible. In section 4.3 we also discussed a possible counterargument against our compatibility claim, Van Inwagen's replay argument. We showed that that argument cuts no ice with us, since the internal stochastic dynamics of the PS model provides an adequate representation of decision finding under indeterminism.

Throughout our discussion, we have tried to remain neutral with respect to controversial issues of metaphysics and psychology. We have not invoked any special theory of agent‐causal powers, nor any metaphysically extravagant assumptions. The core of our model is that a random process of deliberation leading to action can in principle ground the sensible and attributable free agency of a learning agent. We do not claim that our account provides a psychologically or neurologically adequate picture of human free agency, though we are interested in attempts to link the theory to the concrete material basis of our own agency. Even if it turns out that the material basis of our own agency does not match our model, we claim that projective simulation provides a framework for the conceptual discussion, and perhaps also for the technological implementation, of indeterministic deliberation in embodied free agents. *
H. J. B. acknowledges support from the Austrian Science Fund (FWF) through the SFB FoQuS F 4012, and from the Ministerium für Wissenschaft, Forschung und Kunst Baden‐Württemberg (AZ: 33‐7533.‐30‐10/41/1). T. M. acknowledges support from the European Research Council (ERC) under the European Community's Seventh Framework Programme (FP7/2007‐2013), ERC Grant agreement nr 263227. H. J. B. and T. M. both acknowledge support from the Templeton World Charity Foundation grant TWCF0078/AB46.

